# Chronic rhinosinusitis with and without nasal polyps: the state-of-the-art of current treatment strategies and future developments

**DOI:** 10.3389/falgy.2025.1714577

**Published:** 2025-12-15

**Authors:** Emanuele Nappi, Valentina Marzio, Francesco Giombi, Giulia Costanzo, Stefania Merli, Selene Nicolosi, Edoardo Cavaglià, Fabio Lodi Rizzini, Serena Traversi, Alessandro Vrenna, Sofia Vassallo, Gian Marco Pace, Carlo Lombardi, Carlo Maria Rossi, Francesca Puggioni, Luca Malvezzi, Giorgio Walter Canonica, Giovanni Paoletti, Enrico Heffler

**Affiliations:** 1 Personalized Medicine, Asthma and Allergy, IRCCS Humanitas Research Hospital, Rozzano, Milan, Italy; 2 Department of Biomedical Sciences, Humanitas University, Pieve Emanuele, Milan, Italy; 3 Otorhinolaryngology Head & Neck Surgery Unit, Casa di Cura Humanitas San Pio X, Milan, Italy; 4 Department of Public Health and Medical Science, University of Cagliari, Cagliari, Italy; 5 First Department of Internal Medicine, Fondazione IRCCS San Matteo, Pavia, Italy; 6 Unit of Allergology and Immunology, ASST Grande Ospedale Metropolitano Niguarda, Milan, Italy; 7 Dipartimento di Scienze Cliniche e Sperimentali—Scuola di Specializzazione in Allergologia e Immunologia Clinica, Università Degli Studi di Brescia/SSD Allergologia Spedali Civili Brescia, Brescia, Italy; 8 Unità Operativa Semplice Allergologia e Immunologia Clinica, ARNS Civico, Palermo, Italy; 9 Otolaryngology Unit, IRCCS Humanitas Research Hospital, Rozzano, Milan, Italy; 10 Departmental Unit of Allergology, Clinical Immunology and Pneumology, Fondazione Poliambulanza, Brescia, Italy; 11 Department of Internal Medicine and Medical Therapeutics, University of Pavia, Pavia, Italy

**Keywords:** chronic rhinosinusitis, nasal polyps, biologic therapies, CRSwNP, CRSsNP

## Abstract

Over the past decade, chronic rhinosinusitis (CRS) management has undergone substantial transformation, shifting from conventional symptom-focused treatments to precision medicine strategies grounded on molecular insights. The introduction of biologic agents has significantly changed the therapeutic landscape for CRS with nasal polyps (CRSwNP), directly addressing key inflammatory pathways and leading to marked reductions in nasal polyp burden, overall disease impact, and corticosteroid use. Concerns regarding long-term effectiveness, financial burden, and accessibility remain unresolved. Advances in the understanding of the mechanisms underlying CRS are paving the way for the development of novel therapeutic strategies, with increasing attention now also being directed toward the phenotype without nasal polyps (CRSsNP), which currently lacks targeted therapies. Despite progress in pharmacologic therapies, surgery remains a fundamental treatment option, with ongoing efforts to standardize surgical approaches and evaluate novel techniques. Optimizing the integration of surgical and medical therapies while expanding access to novel treatments represents a key future goal in CRS care. This review aims to guide researchers and clinicians through the evolving landscape of CRS management, covering the latest evidence on established and emerging therapies, offering practical insights into endotyping, and highlighting important considerations for the management of severe or refractory cases.

## 1 Introduction

**Figure 1:**
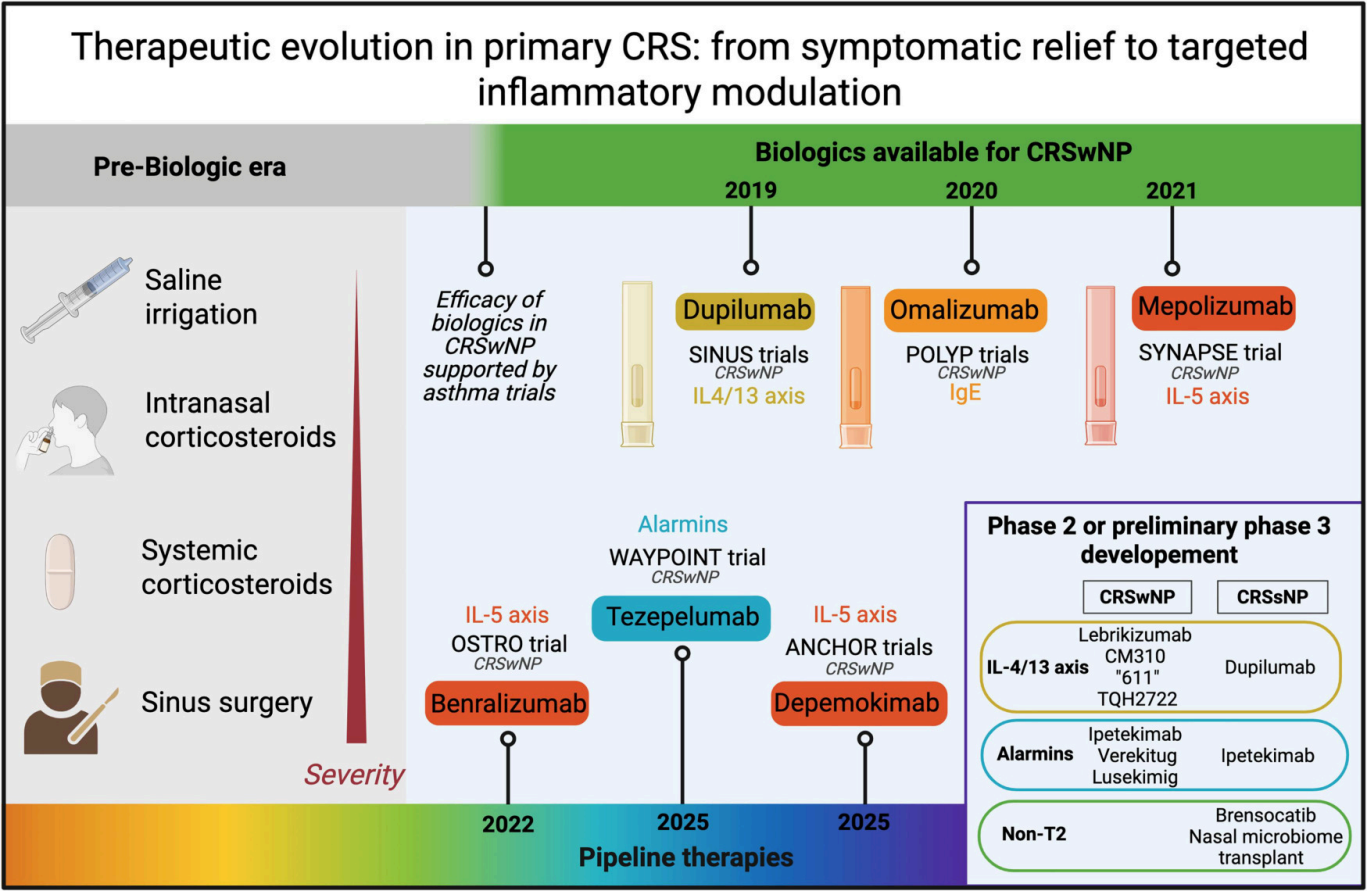
Therapeutic evolution in primary chronic rhinosinusitis (CRS): from symptomatic relief to targeted inflammatory modulation. This figure illustrates the shift from traditional symptomatic therapies (left) toward biologic therapies targeting key inflammatory pathways in CRS. Biologics with completed phase 3 trials to date are shown along the timeline (upper and lower parts of the image). Biologics stemming from the upper timeline (dupilumab, omalizumab, mepolizumab) are currently approved and are regularly used in many countries. Biologics stemming grom the lower timeline (benralizumab, tezepelumab, depemokimab) completed phase 3 DBPCT, but are not yet approved and are under further investigation. Additional drugs under development are presented in the box in the lower right, grouped according to their mechanisms of action. Created with BioRender.com.

Chronic rhinosinusitis (CRS) is a persistent inflammatory condition affecting the mucosa of the nose and paranasal sinuses. CRS is now recognized as a heterogeneous disease influenced by genetic, environmental, microbial, and immunologic factors (1–3). CRS is clinically defined by the presence of sinonasal symptoms lasting for more than 12 weeks, accompanied by objective evidence of mucosal inflammation. CRS is distinguished into primary and secondary, depending on the presence of an identifiable underlining etiology (4). This review is focused on primary CRS, occurring in the absence of an evident cause. Primary CRS has been traditionally classified into two main clinical phenotypes: with (CRSwNP) and without (CRSsNP) nasal polyps, based on endoscopic features (5). The acknowledgment of the critical role of disease endotypes in shaping disease presentation and response to treatment led to a revision of CRS classification in the 2020 edition of the European Position Paper on Rhinosinusitis and Nasal Polyps (EPOS) (6), with further distinctions based on anatomical factors (i.e., localized and diffuse), endotypic factors (i.e., presence or absence of type 2 inflammation), and eventually on phenotypic factors (e.g., presence of polyps, presence of eosinophilic inflammation, allergic fungal rhinosinusitis, and/or central compartment atopic disease). For instance, according to this new classification it is likely that most bilateral CRSwNP cases would fall into the primary diffuse CRSwNP category, whereas most bilateral CRSsNP cases would be subclassified into primary diffuse CRS with or without type 2 inflammation. Figure 1 summarizes the practical steps involved in evaluating and classifying patients with CRS, in line with the EPOS 2020 framework. Nevertheless, the majority of clinical trials on novel CRS therapies still follow the classical phenotypic distinction. Moreover, it is important to note that most of the research on new treatments was focused on the CRSwNP phenotype, possibly because it is more often severe and refractory and because it is strongly linked to type 2 inflammation, making it a target for available biologic therapies that were already available for other type 2-mediated diseases. In more recent years there has been growing interest in exploring the mechanisms and novel targeted therapeutic strategies for other CRS forms as well.

**Figure 2:**
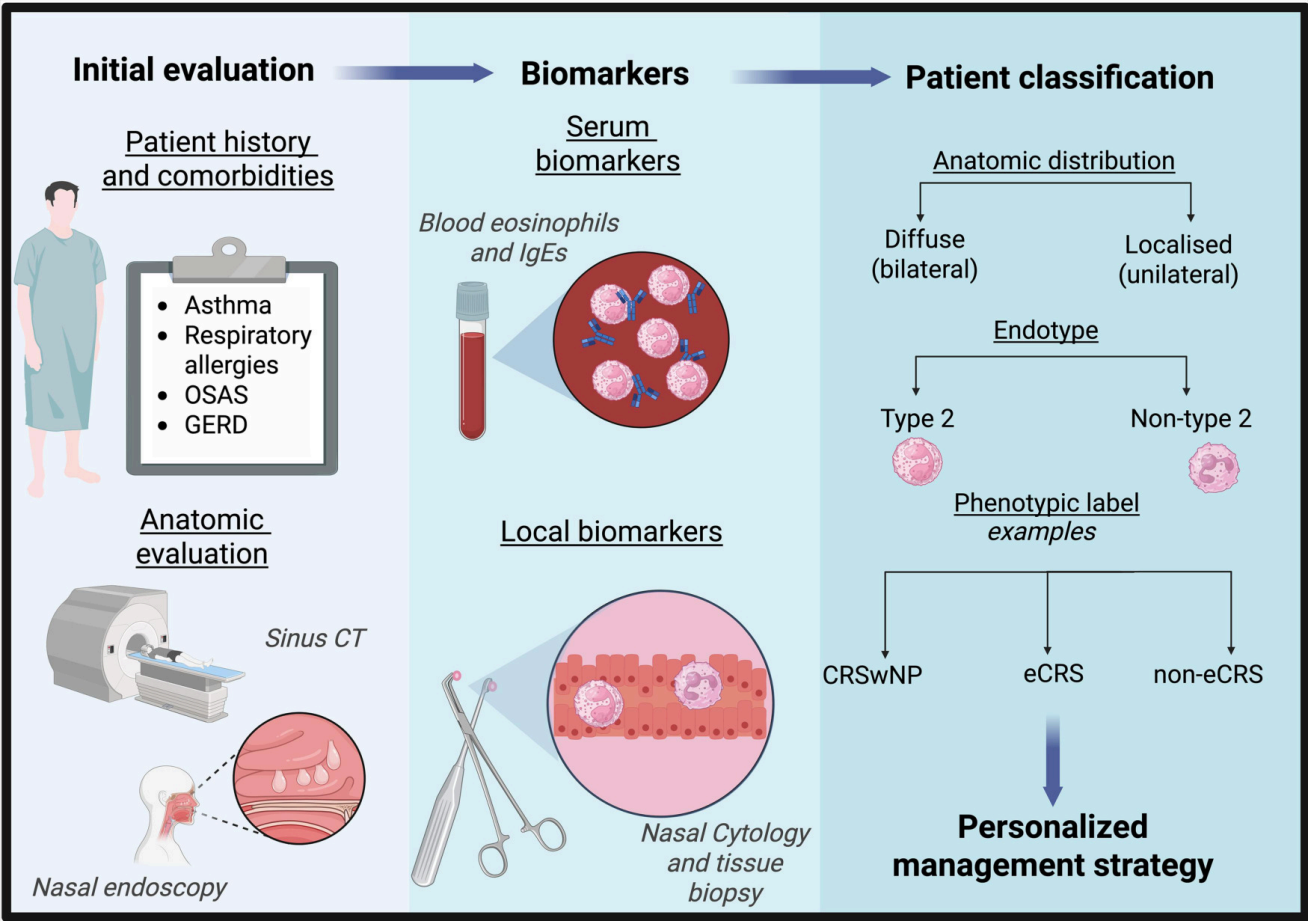
CRS classification in clinical practice. This figure illustrates a practical approach to classifying patients with chronic rhinosinusitis (CRS) in everyday clinical practice. The figure is inspired by the novel classification proposed in the European Position Paper on Rhinosinusitis and Nasal Polyps (EPOS) 2020 by Fokkens W. et al. (1). Objective nasal findings obtained through endoscopy and possibly sinus computed tomography (CT), combined with a thorough assessment of comorbidities, constitute the first step of the evaluation. Serum and possibly local inflammatory biomarkers further refine patient profiling. Together, these components provide the information necessary to assign an appropriate phenotypic label (e.g., chronic rhinosinusitis with nasal polyps – CRSwNP –, eosinophilic CRS – eCRS –, and non-eosinophilic CRS – non-eCRS –) and so to guide a tailored management strategy. OSAS, obstructive sleep apnea; GERD, gastrointestinal reflux disease. BioRender.com

Advancements have led the focus to more refined aspects, including deciphering the inflammatory mechanisms (endotypes) underlying different CRS forms and influencing clinical features and treatment responses (7–9). In the past, CRS treatment primarily relied only on intranasal corticosteroids (INCS), saline irrigation, and antibiotics for secondary infections, with systemic corticosteroids (SCS) and endoscopic surgery implied in refractory cases (10, 11). Even if INCS and surgery continue to be fundamental in CRS management, the identification of specific inflammatory pathways underlining CRS, such as type 2 inflammation, has revolutionized treatment approaches, paving the way for targeted biological therapies (12). In parallel, the therapeutic target of CRS changed, elevating the bar from a barely acceptable symptom control and quality of life (QoL) to clinical remission (i.e., the sustained absence of clinically evident disease manifestations) (13, 14). Figure 2 illustrates the evolution CRS management.

Despite significant progress in CRS treatment, several limitations remain. Currently, biologics are approved only for CRSwNP, with no targeted therapies available so far for CRSsNP. Effective biological therapies for “type-2-low” endotypes are still lacking, underscoring the need for a deeper understanding of CRS pathophysiology to enable precision treatment across endotypes. Moreover, not all patients with type 2 inflammatory CRS respond adequately to biologic therapy; some fail to reach clinical improvement targets (15). Complicating the picture further, in addition to immunologic heterogeneity other factors contribute to the complexity of CRS, including epithelial barrier dysfunction, and microbial dysbiosis (8, 16–18). Future directions in CRS management involve optimizing the integration of surgical and pharmacologic strategies, expanding access to novel treatments, define predictors of response and investigating novel therapeutic agents. This paper provides a comprehensive review of the current state of CRS treatment, highlighting key advancements in both traditional and emerging therapeutic approaches, aiming to inform clinicians and researchers about the evolving landscape of CRS management.

Highlights of this review:

• Updated synthesis of the most recent evidence of currently available therapies, spanning through randomized clinical trials and real-world data evaluating endoscopic sinus surgery, conventional medical treatments, and currently approved biologic therapies for CRS.
• Overview of novel biologic agents and small-molecule drugs under clinical investigation for CRSwNP and CRSsNP, highlighting new molecular targets and potential shifts in future treatment paradigms.
• Practical important considerations for present-day management of refractory CRS and discussion of persisting knowledge gaps and unmet needs to inform future research directions.

## 2 CRS endotypes, biomarkers and therapeutic implications

Advancements in CRS research have underscored the importance of classifying the disease based on its underlying endotypes, particularly in distinguishing type 2, type 1, and type 3 inflammatory pathways, as they present distinct clinical features, comorbidities, and therapeutic responses (19–21). There is no universal way to profile CRS in clinical practice, but such an evaluation may include the combined assessment of numerous factors, most importantly anamnestic, macroscopic, bio-humoral, and microscopic factors.

Briefly, the type 2 endotype, mediated by multiple inflammatory factors (including IL-5, IL-4, IL-13, eosinophilic cationic protein - ECP, and periostin), is characterized by tissue eosinophilia and increased IgE production, associating with asthma, non-steroidal anti-inflammatory drug-exacerbated respiratory disease (N-ERD) and allergic fungal rhinosinusitis (6, 11, 22, 23). Patients within this category generally have a greater disease burden, are difficult-to-treat and tend to recur more frequently and sooner after surgery but can achieve marked improvements with available biologic therapies as they act on type 2 inflammatory pathways (21, 24–27). The type 1 endotype is characterized by a high expression of interferon-*γ* and neutrophilic inflammation, and is clinically characterized by a lower response to corticosteroids (28). Type 3 inflammation, in which IL-17 and IL-22 seem to play a key role, is also associated with a strong neutrophilic response and has been associated with severe, refractory cases of CRS (23). Also the type 3 endotype has a lower responsiveness to corticosteroids, presenting a significant therapeutic challenge and highlighting the need for novel targeted therapies (28). To further complicate the scenario, multiple endotypes frequently coexist in the same patient (20).

Although the type 2 endotype has traditionally been associated with the vast majority of CRSwNP cases in Western populations, numerous studies have shown that it is also frequent among Asian patients. A recent study conducted in southern China reported that type 2 inflammation underlies up to 75% of CRSwNP cases (29). Conversely, type 2 inflammation was long considered uncommon in CRSsNP, however, also this view is being revised. In Europe and North America, the prevalence of the type 2 endotype in CRSsNP has been estimated at approximately 30%–55% (30) whereas it appears less frequent – though not negligible – in Asian patients with CRSsNP. A multicentric study evaluating the cytokine milieu in CRS sinonasal tissue highlighted that it differs markedly across geographic regions (31). Focusing on CRSsNP, IL-5 expression was detected in 36% of CRSsNP patients in Beijing, but only in 5% of those from Chengdu (31).

Distinguishing CRS endotypes and phenotypes allows for an individualized treatment approach, ensuring that medical and surgical interventions are tailored to the specific inflammatory mechanisms, ultimately improving disease management and long-term patient outcomes (6, 7). In research settings there are multiple tools available for a detailed profiling of CRS endotypes, spanning from multiplex assays to quantify numerous protein biomarkers, to mass cytometry for a detailed immune-cell phenotyping, and single-cell RNA sequencing to resolve individual cell populations in the nasal mucosa (32–34). However, in everyday clinical practice the tools available for CRS endotyping are decidedly narrower: clinicians typically rely on clinical features (i.e., comorbidities such as asthma and N-ERD which associated with the type 2 endotype) along with blood eosinophil and total IgE levels and, when available, a standard histologic evaluation of the nasal mucosa. According to the EPOS 2020, the evidence of type 2 inflammation in CRS should be supported by the presence of ≥10 eosinophils per high-power filed (HPF) on nasal biopsies, ≥250 eosinophils/µL on peripheral blood, or ≥100 IgEs/kUI on peripheral blood (6). While these parameters offer a useful framework, they fail to properly characterize all patients (20, 35–37). A clinically applicable tool to improve CRS endotyping is nasal cytology, which enables the direct quantification of inflammatory cells of the nasal mucosa. Nasal cytology is inexpensive, rapid, and easy to perform at the point of care, and it is gaining interest as a method for profiling inflammatory patterns in nasal diseases (38, 39). Several studies demonstrated the potential role of nasal cytology in CRS endotyping, especially in CRSwNP (40–42). The main barriers to the widespread implementation of nasal cytology appears to be the absence of methodological standardization, however, a reproducible and validated methodology for the collection, staining, and interpretation of specimens has indeed been established (43, 44). Furthermore, the methodology has been refined specifically for patients affected by CRSwNP; a clinical study highlighted that tissue eosinophilia identified through nasal cytology provides comparable results to those obtained with histology, with the best accuracy observed with cytological samples taken directly at the level of the nasal polyp (rather than the inferior turbinate which is the standard collection site) (40). From a future perspective, the integration of locally sampled biomarkers, analyzed through accessible techniques, could improve patient stratification. For example, the development of low-cost, point-of-care assays for detecting cytokines or other inflammatory mediators in nasal secretions holds significant potential to advance precision endotyping in routine clinical settings (20).

## 3 CRS management

The management of primary CRS spans through pharmacological and surgical approaches. One size does not fit all, and this stems from the heterogeneity of CRS and the complexity of distinct phenotypes and endotypes. An accurate pretreatment evaluation is crucial to choose the option that most likely will be beneficial for each patient (6). Patients with primary CRS should be initially managed through conventional pharmacologic measures (see next section) and, if these fail, other treatment options (i.e., sinus surgery and biologic therapy) should be considered (45). Both the CRSwNP and the CRSsNP phenotype are frequently accompanied by comorbidities (e.g., asthma, allergic rhinitis, obstructive sleep apnea, immunodeficiencies, gastrointestinal reflux disease and many others) that might be impacted by CRS and at the same time might have an influence on CRS treatment outcomes (46, 47). In this context the interplay between upper and lower airway inflammatory diseases are extremely relevant for patient management (48, 49). Altogether this underscores importance of a multidisciplinary evaluation to properly address both local sinonasal pathology as well as other local or systemic contributors of inflammation (50–52).

### 3.1 Conventional pharmacological therapies

The initial management of primary CRS is pharmacological and involves the use of saline irrigations and topical therapies, most importantly INCS (6). INCS are a cornerstone of CRS therapy due to their potent anti-inflammatory and anti-edematous properties, with minimal systemic and local side effects, even when implied in the long term (53, 54). INCS are potentially beneficial in all primary CRS endotypes, although a lower efficacy has been observed in patients with a high degree of neutrophilic inflammation (28). Their use is associated with polyp size reduction, lower post-surgical recurrence rates, and improved QoL (55). Moreover, INCS play an important role in the post-surgical management of CRS patients, reducing the risk of disease recurrence after endoscopic sinus surgery (ESS) (56); on the other side, endoscopic sinus surgery markedly improves INCS distribution in the nasal mucosa (57). Systemic corticosteroids (SCS), while remarkably effective in alleviating mucosal inflammation, edema and overall symptoms, require cautious use due to significant systemic side effects which contraindicate their use in the long term (6, 58). Consequently, SCS should only be prescribed for short periods in the occasion of disease flares unresponsive to topical treatments. For instance, 1–2 annual short SCS courses of systemic steroids can be used as an adjunct to nasal corticosteroids for patients with uncontrolled or partially controlled disease. In contrast, when a greater exposure to SCS is required to obtain disease control, other approaches should be considered. Systemic antibiotic therapies are used to treat acute infectious exacerbations of CRS (6). In this context, it is important to keep in mind that antibiotic usage rises concerns on side-effects and on the development of antimicrobial resistance. The long-term use of antibiotics in CRS has been object of numerous studies, with a high degree of heterogeneity and variable quality, some of which suggesting positive results (59). Especially in the past, there was a tendency to endorse the use of macrolide antibiotics in CRS, particularly for CRSsNP (59, 60). A DBPCTs on CRSsNP patients treated with daily roxithromycin showed an initial improvement of symptoms and inflammatory biomarkers compared to placebo, especially in patients with low IgE levels; importantly, this improvement waned over time (61). Another DBPCT evaluated macrolide antibiotics in CRS patients (with or without nasal polyps), but with a remarkably distinct posology: azithromycin administered once weekly for 3 months (62). This study did not demonstrate benefits during the treatment period, but 50% of patients experienced symptom improvement 12 weeks afterwards (62). On the other hand, a three-arm randomized placebo-controlled study (MACRO) comparing long-term clarithromycin vs. sinus surgery in patients with CRS (with or without nasal polyps) demonstrated that symptom control at 6 months from randomization was significantly better in the surgery arm, while no significant difference was observed between the placebo and the antibiotic arm (63). To conclude, it might be that antibiotics play a beneficial role in specific CRS endotypes, however, the evidence available at this time does not equivocally support that long- or short-term use of antibiotics impacts on CRS outcomes, more research is needed to support the use of antibiotics in CRS (6, 59, 64–68).

### 3.2 Surgical management

Surgery continues to be a cornerstone of CRS management and should be considered in cases when conventional medical therapies fail to obtain disease control (6). However, uncertainty remains on what defines a sufficient course of medical treatment and thus there are no specific criteria to define when standard medical treatment should be considered unsuccessful (69). Failure of daily high-volume nasal irrigations and INCS, along with short-term SCS courses, generally calls for other interventions, namely sinus surgery and/or targeted therapy. Adherence to topical therapy is an issue and should be addressed before taking further steps (70).

In the past, surgical approaches focused primarily on removing diseased tissue without necessarily restoring normal sinonasal function, possibly deeply altering sinonasal anatomy with long term consequences. However, since the endoscopic technique was first introduced by Stammberger in 1986 the goal of surgery has been to restore proper sinus ventilation promoting the physiological mucociliary clearance (71–73). Functional endoscopic sinus surgery (FESS) is now the gold standard surgical approach for primary diffuse CRS forms and is based on a “functional” criterion grounded in rigid anatomical and physiological principles, specifically, the creation of sinonasal cavities that include the natural ostia to support physiological mucus drainage patterns (6). The preservation of the nasal mucosa and key anatomical landmarks (such as the middle turbinate) is crucial to ensure adequate ciliary function. In this way, sinus surgery is not viewed as a stand-alone procedure but as a complementary approach to enhance the effectiveness of topical medical therapy, which should be continued afterwards. In patients affected by primary CRS, FESS is adequate when it includes anterior and posterior ethmoidectomy, large middle meatal antrostomies, sphenoidotomy and frontal opening (“Full-FESS”) (6).

Importantly, there is a substantial heterogeneity in the surgical approaches employed for CRS, especially in the past. Theoretically, sinus surgery encompasses a broad range of procedures, including alternatives to functional endoscopic techniques that are less invasive but generally less effective, such as balloon sinus dilation and nasal polypectomy. Even within FESS, significant variability exists in the extent of surgery, ranging from limited interventions involving a few sinus chambers to more comprehensive procedures addressing multiple sinuses; only more recently the adequacy of FESS was defined. This variability in surgical completeness has prompted the development of classification systems such as the Amsterdam Classification on Completeness of Endoscopic Sinus Surgery (ACCESS), aiming to standardize the description and evaluation of surgical interventions (74). In parallel, inconsistencies in reporting surgical outcomes (e.g., symptom scores, disease parameters) have further hindered the comparability of results across studies (75). In light of these challenges, evidence concerning the effectiveness of FESS in reducing CRS symptoms and improving QoL is robust (76, 77). Surgery appears to be especially effective in improving nasal obstruction, and less on loss of smell, headache, and post-nasal drip and appears to improve also mucociliary clearance and topical drug delivery to paranasal sinuses (78–80). Furthermore, patients with nasal polyps or comorbidities (i.e., asthma) have demonstrated better improvement of QoL related symptoms immediately after FESS (81). Long-term recurrence rate after surgery range around 14%–60% and 15.5% for CRSwNP and CRSsNP, respectively (76, 82). Clinical factors most consistently associated with disease recurrence after surgery include the presence of nasal polyps, elevated eosinophil counts—particularly in local tissue—the presence of asthma and/or NERD as comorbid conditions, and prior evidence of recurrence following FESS, and time between previous surgery and relapse (25, 26, 83–86). More recently also atopy and familiarity for CRS were linked to a higher risk of recurrence (84). Overall, recurrence rates following surgery for CRS remain a significant concern, highlighting the importance of alternative treatment approaches that directly target the underlying inflammatory processes of the disease. In recent years, evidence has emerged supporting more extensive surgical approaches in specific cohorts of patients with a high inflammatory load, to reduce the risk of local disease reactivation. As the disease becomes more severe, broader surgical resections may help controlling the disease by creating wider fenestrations of sinonasal ostia thus facilitating the instillation of topical medications. Prolonged disease-free rates have been demonstrated in cohorts undergoing extended approaches compared to FESS, yielding a lower long-term revision surgery rate (87). In this perspective, novel surgical approaches have also been proposed. Reboot surgery, developed by Alsharif et al., has been particularly studied in the context of the type 2 endotype and aims to restore a non-inflammatory state in the epithelium by removing eosinophil-infiltrated mucosa from the paranasal cavities, while sparing parts of the inferior conchae (88). By eliminating the inflammatory environment, healthy mucosa can re-epithelialize the sinus walls, reducing the risk of relapse. Diseased mucosa is cleared from all paranasal sinuses down to the periosteum, with careful attention to sensitive areas such as the carotid sheath and optic nerve. According to published series, this approach has shown promise in improving recurrence-free survival and quality of life in patients with recalcitrant CRSwNP (88, 89). Moreover, a recent study involving light and electron microscopy of nasal biopsies before and after reboot surgery of patients affected by CRSwNP demonstrated that the mucosa, including ciliary structures, progressively return to their normal form in the post-operative period (90). Future directions in CRS surgical management are expected to emphasize the integration of surgical intervention with targeted medical therapies, aiming to reduce surgical invasiveness while optimizing the efficacy of post-operative adjuvant treatments (6).

### 3.3 Biologic therapies and other targeted treatments

Monoclonal antibodies (biologic therapies) implied in severe asthma collaterally yielded improvements of upper respiratory symptoms in patients with concomitant CRSwNP (91). From this insight, along with a better understanding of CRS endotypes, researchers developed clinical trials tailored to establish the efficacy of monoclonal antibodies, specifically in CRSwNP. The treatment of CRSwNP has been indeed radically changed since 2019, when the first monoclonal antibody, dupilumab, was approved for severe CRSwNP. Biologic therapies are currently indicated in patients who fail to obtain disease control despite conventional management, including high-volume saline nasal irrigations, INCS, and FESS. The EUFOREA/EPOS 2023 indications for biologic treatment in patients with bilateral CRSwNP already subjected to sinus surgery require the presence of ≥3 of the following criteria: evidence of type 2 inflammation, need for systemic corticosteroids to control disease, significantly impaired QoL, significant olfactory loss and comorbid asthma that requires maintenance inhalatory corticosteroid treatment (45). Type 2 inflammation can be identified with serum and/or tissue biomarkers, as discussed in Section 2. The impact on QoL can be evaluated through the sino-nasal outcome test 22 (SNOT-22), where scores ≥40 indicate a significantly impaired QoL. Olfactory loss should be ideally evaluated with objective testing (cutoff scores vary depending on the specific test implied). As it concerns SCS use, more than 2 cycles per-year or chronic use (even low dose) for more than three months indicates that SCS are needed to control disease (45).

Currently approved biologic therapies for CRSwNP (dupilumab, mepolizumab and omalizumab) target type 2 inflammatory pathways. Clinical trials have in fact demonstrated that these treatments tend to be more effective in patients exhibiting type 2 inflammatory biomarkers, however, they also show therapeutic benefits in broader patient populations, and their use in clinical practice is not strictly limited to patients with an identified type 2 inflammatory endotype (15, 45). Numerous other emerging therapies for CRS - including for “type-2-low” CRS and for CRSsNP in general, which currently lack targeted treatment options - are currently under investigation.

Table 1 summarizes currently approved and investigational therapies for CRS.

**Table 1:** Targeted therapies for CRS.

Drug name	Molecular target	Pivot trials	Achieved outcomes
Dupilumab	IL-4R*α*	SINUS-24 & SINUS-52	Change from baseline to week 24 in NPS and nasal congestion score
Stapokibart	IL-4Rα	CROWN-2	Change from baseline to week 24 in NPS and nasal congestion score
THQ2722	IL-4Rα	NCT06089278	Ongoing
GR1802	IL-4R	NCT06516302	Change from baseline to week 24 in NPS and nasal congestion score.
611	IL-4R	NCT06639295	Ongoing
Lebrikizumab	IL-13	NCT06338995	Change from baseline to week 24 in nasal congestion score and in NPS
Omalizumab	IgE	POLYP-1 & POLYP-2	Change from baseline to week 24 in NPS and average daily nasal congestion score.
Mepolizumab	IL-5	SYNAPSE & MERIT	Change from baseline in total endoscopic NPS at week 52 and nasal obstruction VAS (weeks 49–52)
Benralizumab	IL-5R	OSTRO	Change from baseline to week 40 in NPS score and patient-reported mean nasal blockage score once every 2 weeks.
Depemokimab	IL-5	ANCHOR-1 & ANCHOR-2	Change from baseline in total endoscopic NPS at week 52 and mean nasal obstruction score (weeks 49–52).
Tezepelumab	TSLP	WAYPOINT	Change from baseline to week 52 in total NPS and bi-weekly mean nasal congestion score.
Ipetekimab	IL-33	NCT06834347	Ongoing
Verekitug	TSLP	NCT06164704	Ongoing
Lunsekimig	TSLP & IL-13	NCT06454240	Ongoing

This table summarizes targeted therapies for CRS, spaning trhough currently approved therapies and drugs under investigation. TSLP: thymic stromal lymphopoietin; NPS: nasal polyp score; SNOT-22: sinonasal outcome test 22.

#### 3.3.1 Biologic therapies acting on the IL-4/IL-13 axis

**Dupilumab** is a monoclonal antibody that targets both IL-4 and IL-13, blocking the subunit alpha of the IL-4 receptor and thus signaling of the IL- 4 and IL-13 cytokines, which are critical players of type 2 inflammation (92). Dupilumab is approved for the treatment of CRSwNP, severe asthma, chronic obstructive pulmonary disease with high eosinophils, atopic dermatitis, prurigo nodularis and eosinophilic esophagitis. The approval for CRSwNP was based on the results from LIBERTY NP SINUS-24 and LIBERTY NP SINUS-52 studies (93). These randomized double-blind placebo-controlled trials (DBPCT), along with subsequent studies, also in real-life settings, well-demonstrated the overarching efficacy of dupilumab on important CRS parameters, among which the nasal polyp score (NPS), the SNOT-22 (including the olfactory parameters), reducing the need of SCS and of subsequent sinonasal surgery (93–95). Patients with CRSwNP and concomitant asthma seemed to have an even greater improvement of CRS outcomes with dupilumab than the ones without asthma. Real life experiences (96–98) confirmed DBPCT findings, highlighting significant changes in all outcomes measured within just 1 month of treatment, with improvements in inflammatory biomarkers and lung function just 2 weeks after treatment initiation (99, 100). As for olfactory dysfunction, dupilumab improved smell function regardless of prior surgery, comorbid asthma, or N-ERD (99, 101). Focusing on lung function, a retrospective study on a cohort of German patients highlighted the improvement of the FEV1 in patients with CRSwNP during dupilumab treatment (102). This finding was previously seen also in the *post hoc* analyses of the SINUS 24 and 52 studies and of the QUEST study (103, 104). Dupilumab has a good safety profile, but it raised some concern due to the frequently observed increase in peripheral eosinophil levels, asymptomatic in the vast majority of cases; this particular aspect as well as the safety of biologic therapy in CRS in general is addressed in Section 3.3.4. More recently, dupilumab is being studied also in CRSsNP. Results from the ORION trial (NCT04678856), a phase 3 DBPCT on CRSsNP irrespective of eosinophil levels, preliminarily published in the form of abstract, indicate that dupilumab significantly reduces the computed tomography Lund-Mackay score in CRSsNP patients with peripheral eosinophil levels above 300 cells/µL, with numerical reductions also in olfactory and symptom parameters (105). A subsequent study investigating the role of dupilumab in severe eosinophilic CRSsNP is planned (NCT04430179).

Beyond dupilumab, other drugs acting on the IL4/IL13 axis are under study also in the CRS field. Stapokibart, a monoclonal antibody directed to the alpha subunit of the IL-4 receptor, has demonstrated efficacy in significantly reducing nasal congestion and NPS in a phase 3 DBPCT (CROWN-2) in a Chinese study on patients with severe CRSwNP (106). The NPS reduction was numerically higher in patients with eosinophilia. Another biologic that acts on the same target, TQH2722, is under study for atopic dermatitis and CRS; so far, a phase 2 DBPCT on CRS patients with or without nasal polyps has been completed in April 2025 (NCT06089278), with results not yet available, and another phase 2 with a longer study period is now recruiting (NCT06439381). Additional drugs that act on the IL4/13 axis are under current investigation in CRS, including; GR 1802, an anti-IL-4 receptor (NCT06516302 – phase 3, not yet recruiting); “611” an anti-IL4 receptor (NCT06639295 – phase 3, not yet recruiting); and lebrikizumab, an IL-13 inhibitor (NCT06338995 – phase 3, recruiting).

#### 3.3.2 Biologic therapies acting on igEs

**Omalizumab**, a monoclonal antibody that targets IgEs, received FDA approval for the treatment of CRSwNP in 2020. Data from DBPCTs showed that omalizumab improves CRSwNP outcomes, with an excellent safety profile, irrespective of coexisting allergies, asthma, or levels of IgE or blood eosinophil count (107, 108). Consequently, a broader spectrum of CRSwNP patients seem to benefit from omalizumab therapy than simply the ones with IgE mediated respiratory allergies. A meta-analysis provided evidence that omalizumab is also capable of reducing the need for sinus surgery in CRSwNP patients, but not of systemic corticosteroids (109). A real–world study on 27 patients with CRSwNP treated with omalizumab confirmed its effectiveness, which seemed to be even higher in males and in those with a type 2 inflammatory endotype (110).

#### 3.3.3 Biologic therapies acting on the IL-5 axis

**Mepolizumab** is a monoclonal antibody that targets and neutralizes IL-5, inhibiting eosinophilic inflammation. It was first approved for the treatment of severe eosinophilic asthma and subsequently it obtained authorization for eosinophilic granulomatosis with polyangiitis (EGPA), hypereosinophilic syndrome and CRSwNP. Retrospective data from cohorts of patients with asthma who started mepolizumab between 2014 and 2021 suggested short- and long-term efficacy of mepolizumab in CRSwNP in real word settings (111). In 2021, mepolizumab received FDA approval as the third biologic for CRSwNP based on the results of the SYNAPSE randomized DBPCT, which demonstrated that in patients with CRSwNP mepolizumab leads to a significant reduction in CRS symptoms, NPS and in the necessity for oral corticosteroids and sinus surgery. A subsequent DBPCT (MERIT) evaluated mepolizumab in Japanese, Chinese, and Russian patients with eosinophilic CRSwNP. The study demonstrated significant improvement in nasal obstruction, as well as a reduction in NPS which, however, did not reach statistical significance. Nevertheless, this more modest change in NPS was accompanied by meaningful gains in QoL and a decreased need for corticosteroids and sinus surgery (112). The effectiveness of mepolizumab in CRSwNP is also supported by real world experiences: mepolizumab significantly lowered symptoms severity and size of polyps irrespective of comorbid N-ERD, prior surgery, allergic rhinitis, or systemic corticosteroid use. A recent paper corroborates existing findings, demonstrating that mepolizumab was effective, well-tolerated, and safe for treating CRSwNP, reduced the necessity for SCS and surgical interventions, and improved QoL (113). Further investigations are ongoing, for example the RESPONSE study, a real-world, prospective cohort research aimed at evaluating the efficacy of mepolizumab in adult patients with concurrent severe asthma and CRSwNP.

**Benralizumab** is an anti-IL5 receptor monoclonal antibody approved for the treatment of severe eosinophilic asthma, which also showed positive results in EGPA. Benralizumab is under investigation for CRSwNP, but it currently lacks approval. The phase 3 OSTRO DBPCT showed that benralizumab significantly decreased NPS, nasal congestion score, and improved olfactory function, however it failed to achieve a statistically significant improvement in QoL as evaluated by the SNOT-22 (114). A DBPCT to further evaluate the efficacy of benralizumab in this context is ongoing and is expected to terminate by April 2025 (NCT04157335). Parallelly, a recent real-world study revealed that 12 months of benralizumab therapy yield improvements in nasal polyp burden and asthma outcomes in patients with severe eosinophilic asthma and concomitant CRSwNP (115).

**Depemokimab** is an anti-IL-5 monoclonal antibody that has the peculiarity of having a very long half-life and a high affinity for IL-5, enabling administration only twice per year. It is under investigation for asthma, EGPA and CRSwNP. Results from the phase 3 DBPCTs ANCHOR-1 and ANCHOR-2 of depemokimab in CRSwNP have just been published, demonstrating that it significantly reduces NPS and nasal obstruction, but it did not significantly change SNOT-22, olfactory impairment, as well as the need for surgery and for “disease-modulating medications”, including SCS (although comparison was hindered by the relatively low rate of these latter two events in both groups) (116, 117).

#### 3.3.4 Biologic therapies acting on alarmins involved in type 2 inflammation

**Tezepelumab** is a monoclonal antibody that inhibits the alarmin thymic stromal lymphopoietin (TSLP) and was recently approved for the treatment of severe asthma. Tezepelumab acts upstream in the type 2 inflammatory cascade with respect the other previously mentioned drugs that act on type 2 inflammation; this rose potential concerns on safety, which however has not been confirmed by clinical studies (118). The phase 3 DBPCT WAYPOINT on tezepelumab in CRSwNP showed significant improvements in nasal polyp score, symptom scores (including olfaction and nasal congestion), SNOT-22 while reducing the need for SCS and sinus surgery (119). The role of tezepelumab in CRSwNP is being furtherly investigated through a phase 4 ESSENCE trial (NCT06706817), currently in the recruitment phase.

Concerning other investigational therapies that act at the alarmin level, ipetekimab is a monoclonal antibody directed at the alarmin IL-33 that is being investigated in type 2 inflammatory diseases, including moderate-to-severe asthma. Promising results in this context fostered research in CRS as well: ipetekimab is now under investigation through phase 3 trials in CRSwNP (NCT06834347, NCT06834360), and also in CRSsNP through a proof-of-concept DBPCT (NCT06691113), all of which are currently recruiting. Along this line of research, phase 2 trials are investigating the role of verekitug (NCT06164704), an anti-TSLP receptor, and lunsekimig (NCT06454240), a bispecific anti-TSLP and -IL-13, in CRSwNP. Other biologics that act at the alarmin level are under study for asthma (e.g., anti-IL-33: etokimab, itepekimab; anti-IL-33R: astegolimab, melrilimab), but there are no clinical trial in CRS to date.

#### 3.3.5 Investigational therapies acting on non-type 2 targets

Brensocatib is an orally administered small molecule that interferes with neutrophil activity through the inhibition of a neutrophil serin-protease. This drug has been initially investigated in non–cystic fibrosis bronchiectasis (120). Brensocatibis being studied in CRSsNP through the phase 2 BiRCh trial (NCT06013241), which was concluded in August 2025, with results not yet available.

An emerging area of research is centered on the nasal microbiome. Growing evidence indicates that shifts in microbial composition and diversity, as well as the presence of specific bacterial species, may play a role in CRS pathogenesis, especially in CRSsNP (121, 122). Moreover, microbiome transplantation is effective in other settings, notably Clostridioides difficile colitis. Altogether this brought to the development of clinical trials exploring the potential therapeutic value of microbial transplants in CRS. A small non-randomized clinical study on patients with CRSsNP subjected to nasal microbial transplant from healthy donors administered for 5 consecutive days and after a course of antibiotic treatment showed significant improvements in SNOT-22 along increased microbial abundance and diversity, but not in total nasal symptoms scores nor on endoscopic parameters (123). In addition, a more recent case series evaluating the role of nasal microbiota transplant without prior antibiotic therapy demonstrated symptom improvement in 2 out of the 3 cases, with benefits up to 6 months after the procedure. A phase 2 DBPCT investigating the role of microbiome transplant in CRSsNP is currently recruiting with an expected completion by December 2025 (NCT05400616).

### 3.4 Important considerations on CRS management

#### 3.4.1 Current lack of targeted treatment options for “type-2-low” CRS and for CRSsNP

Biologic drugs currently available for CRS have been studied and approved in patients with nasal polyps. While significant progress has been made in targeting type 2 inflammation in CRSwNP, much less effort has been dedicated in the development of novel treatments for CRSsNP (124). This disparity might be attributed both to the generally greater symptom burden associated with CRSwNP (125), prioritizing it as a treatment target, and to the strong association between CRSwNP and the type 2 endotype, making it a suitable target to biological therapies already available, in contrast with the marked heterogeneity of CRSsNP which may pose greater challenges for research in this field. The recognition that the type 2 endotype is also frequently present in CRSsNP (20, 23) combined with a growing understanding of the inflammatory alterations in “type-2 low” patients is fostering research efforts in CRSsNP both for type-2-targeted interventions and for therapies acting on other inflammatory pathways. Given the current unavailability of targeted pharmacologic interventions for refractory CRSsNP cases in common clinical practice, sinus surgery has a pivotal role in management. Although macrolide strategies have been endorsed, and can be beneficial in some patients, the most recent evidence on this topic does not support their use, especially in favor of surgery, as thoroughly discussed in the conventional therapies section. When even surgery fails, participation to clinical trials on novel CRSsNP treatments should be considered.

#### 3.4.2 Treatment response and remission

Although studies have demonstrated early benefits soon after treatment initiation, the maximal benefit might not be appreciated up to months after the initiation of biologic therapy. The response to biologic therapy, and thus the decision on whether or not to continue to pursue that treatment strategy, should be evaluated at 6 months and then again at 12 months after initiation (45). The parameters that should be considered in evaluating treatment efficacy are nasal polyp size, QoL, olfaction and the need of SCS to obtain disease control; if comorbidities (e.g., asthma) are present, also the improvement in comorbidities should be considered an outcome parameter (45). Historically, CRS management focused primarily on symptom control, but the introduction of more effective therapies led to a revision of treatment objectives towards achieving sustained disease remission rather than just disease control. This shift parallels progresses in other type 2 inflammatory conditions, such as asthma and eosinophilic esophagitis, where remission has become the therapeutic target (13). According to a recent consensus statement of the EPOS and European Forum for Research and Education in Allergy and Airway disease (EUFOREA), remission in CRSwNP is defined as the sustained absence of significant symptoms, no need for sys lack of active disease on endoscopy for at least 12 months (14). Experts proposed that both patient-reported outcomes (e.g., SNOT-22, VAS) and objective tools as nasal endoscopy should be considered, however, precise cut-offs for defining CRS control and remission are yet to be fully defined and validated, and this highlights a current gap in CRS management (13, 14). While waiting for criteria to be defined, absence of relevant CRS symptoms could be defined as a SNOT-22 <20 as suggested by data on healthy subjects (126). Olfaction, a particularly impactful symptom, should be specifically evaluated, ideally with objective tests indicating isosmia or, alternatively, a self-reported VAS on olfactory loss; in the latter case precise cutoffs are lacking, but values ≤3.5 should be acceptable (127). Concerning objective measures, whether the absence of endoscopic activity necessarily requires nasal polyps to be completely regressed (i.e., NPS = 0) and/or if other endoscopic parameters should be taken into consideration (e.g., nasal secretions, edema) is a matter of debate and yet to be defined.

#### 3.4.3 Comparative effectiveness and treatment failure

Although biologic therapy is effective in most patients, a relevant proportion might not obtain disease control with available treatments. This variability might reflect the underlying heterogeneity of type 2 inflammation itself, with different patients having distinct dysregulated components of the type 2 cascade, highlighting the critical need for deeper insights into type 2 mechanisms and the development of predictive biomarkers to identify individuals most likely to benefit from specific therapies. Real word-data suggests that the percentage of CRSwNP patients that has a very good response to biologic therapy approximates 60% (128). Awaiting definitive evidence from head-to-head trials, observational studies suggest a possibly greater efficacy for dupilumab over other currently approved anti-IL-5 and anti-IgE therapeutic options (129). Moreover, pooled analyses of distinct DBPCTs evaluating the efficacy of biologics for CRSwNP also show a possible superiority of dupilumab over other biologics (130). Although these are precious insights, it is important to note that these studies designs do not prove definitive evidence of treatment superiority, and head-to-head trials should be used as a reference to evaluate this outcome. Notably, the trials showed a wide heterogeneity with respect to (i) participants' baseline type 2 inflammatory profile, (ii) eligibility criteria for inclusion (e.g., NPS and SNOT-22 thresholds, prior SCS use), (iii) outcome measurement and definition, and (iv) co-utilization of INCS throughout the study period. These variability hampers comparability across study findings and warrants a cautionary approach when interpreting results from distinct studies.

Recently, results from the first head-to-head randomized controlled trial in this context (EVEREST) were published. The EVEREST study compared the efficacy of omalizumab vs. dupilumab in patients affected by severe CRSwNP and concomitant asthma, showing that dupilumab is superior to omalizumab for all primary and secondary endpoints, with a difference in NPS reduction from baseline of 1.6 points (greatest reduction patients treated with dupilumab) after 6 months of treatment (131). Furthermore, a real-world, head-to-head study of dupilumab vs. mepolizumab in Danish CRSwNP patients (NCT05942222), aiming at evaluating the non-inferiority and potential superiority of dupilumab compared to mepolizumab, is ongoing with a conclusion foreseen for March 2026.

In the setting of biologic therapy failure its necessary to re-evaluate the patient to define whether to interrupt treatment and in that case if it is most appropriate to pursue with surgical procedures or medical treatment with another biologic agent (45). Patients who fail a biologic treatment might benefit from switching to a different biologic. Real-life experiences have shown CRSwNP outcomes improvement with dupilumab in patients not responding to omalizumab or mepolizumab and vice-versa (132, 133). A Canadian multicenter experience reported at least one switch in 16% patients undergoing biological treatment (134). Adverse events were the leading reason for switching from dupilumab, while insufficient symptom control was the main cause for switching from mepolizumab and omalizumab. Currently there are no specific criteria on when it is indicated to switch biologic therapy.

#### 3.4.4 Safety, vaccinations and monitoring

The safety profile of biologics in use for CRS is very good, with rate and type of adverse events substantially comparable to placebo in the relative phase 3 clinical trials mentioned above, moreover safety is confirmed also by an analysis of real-world data (135). There is no strong evidence in support of a higher rate of severe infections and no specific infectious monitoring is warranted throughout therapy. As it concerns parasitic infections, a study on a large number of individuals (≈90,000) showed an overall very low rate of parasitic infections on treatment, with no significant difference in the rate of parasitic infections between controls and patients treated with omalizumab, dupilumab and mepolizumab, but with a slightly higher rate in patients with benralizumab (136).

Dupilumab raised concern for the rise in peripheral eosinophil levels observed in DBPCTs, confirmed by real-world cohorts (93, 99, 137). This phenomenon is possibly a side effect of dupilumab which blocks the entrance of eosinophils into tissues, favoring the initial accumulation of eosinophils in the bloodstreams (138). The increase in peripheral eosinophil levels is indeed temporary and tends to decrease throughout treatment, along with total IgE and eosinophilic cationic protein levels (139). The is no evidence that absolute eosinophil cunt is consistently linked to adverse events throughout dupilumab treatment, however, isolated cases of systemic eosinophilic diseases - for which patients with pre-existing CRS and/or asthma may be already at greater risk - occurring under dupilumab treatment have been reported (140, 141). Nonetheless, peripheral eosinophil levels should be measured under dupilumab (e.g., baseline, 1 month and every 3 months at least initially; as for patients who develop hypereosinophilia (absolute eosinophil count >1,500 cells/µL), strict monitoring is warranted, with monthly complete blood count and organ damage assessment (even with supplemental tests if symptoms compatible with eosinophilic organ damage are present), and paying particular attention to patients with eosinophil values above 3,000 cells/µL (138). All this considered, discontinuation of dupilumab in asymptomatic patients with hypereosinophilia is deemed unnecessary.

Although in most countries vaccination with live-attenuated vaccines is discouraged throughout biologic therapy, current evidence indicates that vaccination in patients receiving dupilumab, mepolizumab and omalizumab is safe and does not impair vaccine immunogeneity, including for live-attenuated vaccines (142, 143). As it concerns drugs that act upstream in the type 2 inflammatory cascade (e.g., tezepelumab) or on other immunological targets, vaccine safety and efficacy is yet to be fully evaluated. For tezepelumab, no suppression of the humoral immune response to influenza vaccine was observed, but awaiting for further safety studies, the use of live-attenuated vaccines should be carefully evaluated (144).

#### 3.4.5 Therapy duration and tapering

A critical aspect of biologic therapy is that its efficacy has been demonstrated only during treatment, and DBPC trails on this topic suggested that the disease recurs upon interruption (45). It would be necessary to define how to manage treatment schedules in the long term, especially in patient who are under control. A study observed no significant difference in NPS and nasal congestion or obstruction between patients receiving dupilumab 300 mg every 2 weeks and those undergoing treatment with dupilumab 300 mg every 2 weeks for 24 weeks, followed by dupilumab 300 mg every 4 weeks (145). Furthermore, the CRS control in patients who underwent dupilumab tapering for 2 years was evaluated, demonstrating that the therapeutic efficacy established within 24 weeks lasts during the tapering (145, 146). Regarding mepolizumab, Desrosiers et al. observed sustained clinical benefits up to 24 weeks after discontinuation in patients with severe CRSwNP who underwent 52 weeks of therapy (147). Currently there are no recommendations on how to apply biologic tapering in clinical practice, and further studies are needed to define whether tapering would be a proper strategy to maintain clinical benefits.

#### 3.4.6 Accessibility to novel treatments and economic considerations

Biologic agents represent the major new cost driver in CRS care. Their high price, and the need for prolonged treatment determine overall expense and access. Cost-effectiveness analyses consistently show that currently approved biologics (dupilumab, omalizumab, mepolizumab) improve quality of life and reduce surgery and corticosteroid use, but remain expensive interventions, with evidence indicating that surgery is more cost effective then biologic therapy at current prices (148, 149). Drug price and treatment duration are the main factors influencing cost, with biologic therapy generally considered cost-effective only in patients with severe, uncontrolled disease and clear type-2 inflammation.

Disparities in access to CRS care based on income, race, and health insurance status have been highlighted and are a major issue (150). Furthermore, specialist evaluation and authorization are required to access to second-level treatments (e.g., sinus surgery, biologic therapies, clinical trials) and, in many regions, limited rhinology expertise delays or restricts access.

## 4 Conclusions

The evolving understanding of CRS as a complex, immune-driven disorder has reshaped its management. Personalized care grounded on endotypic and phenotypic profiles is gradually replacing one-size-fits-all strategies—particularly in CRSwNP, where biologics have shown meaningful clinical impact. Yet, progress remains uneven: CRSsNP and “type 2-low” disease still lack effective targeted therapies, and the absence of practical tools to stratify patients in clinical settings limits the implementation of precision medicine in everyday practice. Bridging this gap will require research focused not only on novel treatments, but also on accessible biomarkers and decision-making tools that can inform care in real-world practice.
